# Global maternal health country typologies: A framework to guide policy

**DOI:** 10.1371/journal.pgph.0003867

**Published:** 2024-11-11

**Authors:** Zachary J. Ward, Rifat Atun, Gary King, Brenda Sequeira DMello, Sue J. Goldie

**Affiliations:** 1 Center for Health Decision Science, Harvard T.H. Chan School of Public Health, Harvard University, Boston, Massachusetts, United States of America; 2 Department of Global Health and Population, Harvard T.H. Chan School of Public Health, Harvard University, Boston, Massachusetts, United States of America; 3 Department of Health Policy and Management, Harvard T.H. Chan School of Public Health, Harvard University, Boston, Massachusetts, United States of America; 4 Department of Global Health and Social Medicine, Harvard Medical School, Harvard University, Boston, Massachusetts, United States of America; 5 Institute for Quantitative Social Sciences, Harvard University, Cambridge, Massachusetts, United States of America; 6 Maternal and Newborn Healthcare, Comprehensive Community Based Rehabilitation in Tanzania (CCBRT), Dar Es Salaam, Tanzania; 7 Global Health Education and Learning Incubator, Harvard University, Cambridge, Massachusetts, United States of America; University of Embu, KENYA

## Abstract

Maternal mortality remains a large challenge in global health. Learning from the experience of similar countries can help to accelerate progress. In this analysis we develop a typology of country groupings for maternal health and provide guidance on how policy implications vary by country typology. We used estimates from the Global Maternal Health (GMatH) microsimulation model, which was empirically calibrated to a range of fertility, process, and mortality indicators and provides estimates for 200 countries and territories. We used the 2022 estimates of the maternal mortality ratio (MMR) and lifetime risk of maternal death (LTR) and used a *k*-means clustering algorithm to define groups of countries based on these indicators. We estimated the means of other maternal indicators for each group, as well as the mean impact of different policy interventions. We identified 7 groups (A-G) of country typologies with different salient features. High burden countries (A-B) generally have MMRs above 500 and LTRs above 2%, and account for nearly 25% of global maternal deaths. Countries in these groups are estimated to benefit most from improving access to family planning and increasing facility births. Middle burden countries (C-E) generally have MMRs between 100–500 and LTRs between 0.5%-3%. Countries in these groups account for 55% of global maternal deaths and would benefit most from increasing facility births and improving quality of care. Low burden countries (F-G) generally have MMRs below 100 and LTRs below 0.5%, account for 20% of global maternal deaths, and would benefit most from improving access to family planning and community-based interventions and linkages to care. Indicators vary widely across groups, but also within groups, highlighting the importance of considering multiple indicators when assessing progress in maternal health. Policy impacts also differ by country typology, providing policymakers with information to help prioritize interventions.

## Introduction

Maternal mortality remains a large challenge in global health, with one of the Sustainable Development Goals (SDG) set by the United Nations (UN) to reduce the global maternal mortality ratio (MMR) to less than 70 maternal deaths per 100,000 live births by 2030, with no individual country above 140 [1]. Although progress has been made, the SDG targets are unlikely to be met on current trends [2,3]. Learning from the experience of other countries, both what works and what does not, can help to accelerate improvements in maternal health and contribute to global and country-specific progress towards the SDG targets.

Although countries are often grouped by geographic region, income group, or other socio-demographic indices in an attempt to provide more generalizable insights [2–5], these may not be the most informative groupings for maternal health policy considerations. Countries that may be similar in some respects may differ in terms of what interventions would yield the largest impact on maternal health in each setting, while other countries may have similar maternal health needs but are otherwise different.

An obstetric transition framework has been previously proposed which is specific for maternal health, and groups countries based on levels of MMR [6]. Although this framework can provide insights on levels of obstetric risk among countries, country groupings based on a single indicator of MMR are not sensitive to changes in fertility.

In this analysis, we develop a typology of country groupings that is salient for maternal health and is sensitive to both levels of obstetric risk and changes in fertility. We also examine how other maternal health indicators vary by these country typologies and provide guidance on how maternal health policy implications vary by country typology.

## Methods

We used estimates from the Global Maternal Health (GMatH) microsimulation model, which was empirically calibrated to data on a range of fertility, process, and mortality indicators related to maternal health, and has been shown to have good predictive accuracy [3]. The GMatH model simulates individual women in 200 countries and territories, derived from an exhaustive list of areas from the United Nations, with 37 excluded due to political affiliations with other countries [3]. Bayesian hierarchical models were used for all model parameters, and 1000 simulations were performed with the final calibrated model sampling from the best-fitting 100 parameter sets, accounting for uncertainty (and missing data) for all model inputs. The model provides estimates for a range of maternal health indicators, allowing for a more comprehensive view of maternal health outcomes in different contexts.

In this analysis we group countries using two indicators: the maternal mortality ratio (MMR), defined as the number of maternal deaths per 100,000 live births, and the lifetime risk of maternal death (LTR), which is estimated as the sum of age-specific maternal mortality rates between the ages of 15 to 49 and can be interpreted as the probability that a 15-year old female will die from a maternal cause in her lifetime, holding fertility and mortality risks constant at current levels. The MMR is often used to assess the obstetric risk of death per pregnancy, while the LTR is sensitive to changes in both fertility and obstetric risk and can be used to assess the cumulative risk of repeated pregnancy exposure.

We plotted each country in this two-dimensional space (MMR and LTR), using the mean estimates for 2022 for each indicator, and grouped countries using a *k*-means clustering algorithm, which aims to partition data into a number of clusters such that each observation belongs to the cluster with the nearest mean (i.e., centroid) [7]. For this analysis we specified that countries should be clustered into 7 groups as a balance between parsimony and the ability to distinguish salient features. For each cluster, we estimated the mean MMR and LTR weighted by population size to describe the cluster characteristics. We also estimated the cluster means for other indicators to provide more context: total maternal deaths (including late maternal deaths); total fertility rate (TFR, age-standardized fertility rates calculated among women ages 15–49); contraceptive prevalence rate (CPR, defined as the proportion of women ages 15–49 using any method of contraception); proportion of deliveries that occur in medical facilities (facility births); and the c-section rate (the proportion of deliveries that occur via c-section, both emergency and elective).

We also estimated the mean projected reduction in maternal deaths from 2030–2050 (weighted by population size) for policy strategies (previously modelled) [8] to characterize the policy priorities for each cluster. For policy strategies we considered interventions to scale-up coverage levels of various factors to targets informed by the means among high income countries: family planning (contraception and abortion care [i.e., medical abortion] to improve sexual and reproductive care), community-based interventions and linkages to care (antenatal care, skilled birth attendants for home births, improved referral and transportation to health facilities [i.e., healthcare access]), facility-based interventions and linkages to care (increased facility births, availability of clinical services, and improved linkages to care), and a facility-based intervention that improves the quality of care [8]. Although using the mean among high income countries to inform target coverage levels may be overly optimistic, the advantage of this approach is that it standardizes the scale-up definitions across the various strategies to assess their comparative effectiveness. See Table 1 for a description of the policy strategies and target coverage levels.

**Table 1:** Policy intervention descriptions.

Policy Strategy	Description
Family planning	Contraception: Reduce unmet need for contraception to 20% Medical abortion: Increase proportion of abortions that are ‘safe’ to 95%
Community-based + Linkages to care	ANC: Increase probability of any antenatal care (ANC) visits to 95%, with a minimum number of 4 visits (given any visits) SBA: Increase proportion of home births attended by a skilled birth attendant (SBA, trained medical professional) to 80%, and increase use of clean birth kits to 90% and management of moderate hemorrhage to 50% Referral: Increase recognition and referral of complications from SBA (home) to 90%, non-EmOC to 95%, and BEmOC to 99% Transportation: Increase availability of timely transportation from home to 80%, SBA-Home to 85%, non-EmOC to 97%, and BEmOC to 99% Targeted transfers: Reduce horizontal transfers (i.e., to a facility of the same level) for Non-EmOC to 1% and BEmOC to 10%
Facility births	Increase proportion of facility births to 99%
Quality of care	Improve complication recognition and quality of care at Non-EmOC to 90%, BEmOC to 95%, and CEmOC to 99%

Targets for scale-up were informed by the estimated mean level among high-income countries in 2022. Non-EmOC: Non-emergency obstetric care facilities, BEmOC: Basic emergency obstetric care facilities, CEmOC: Comprehensive emergency obstetric care facilities.

## Results

**Fig 1:**
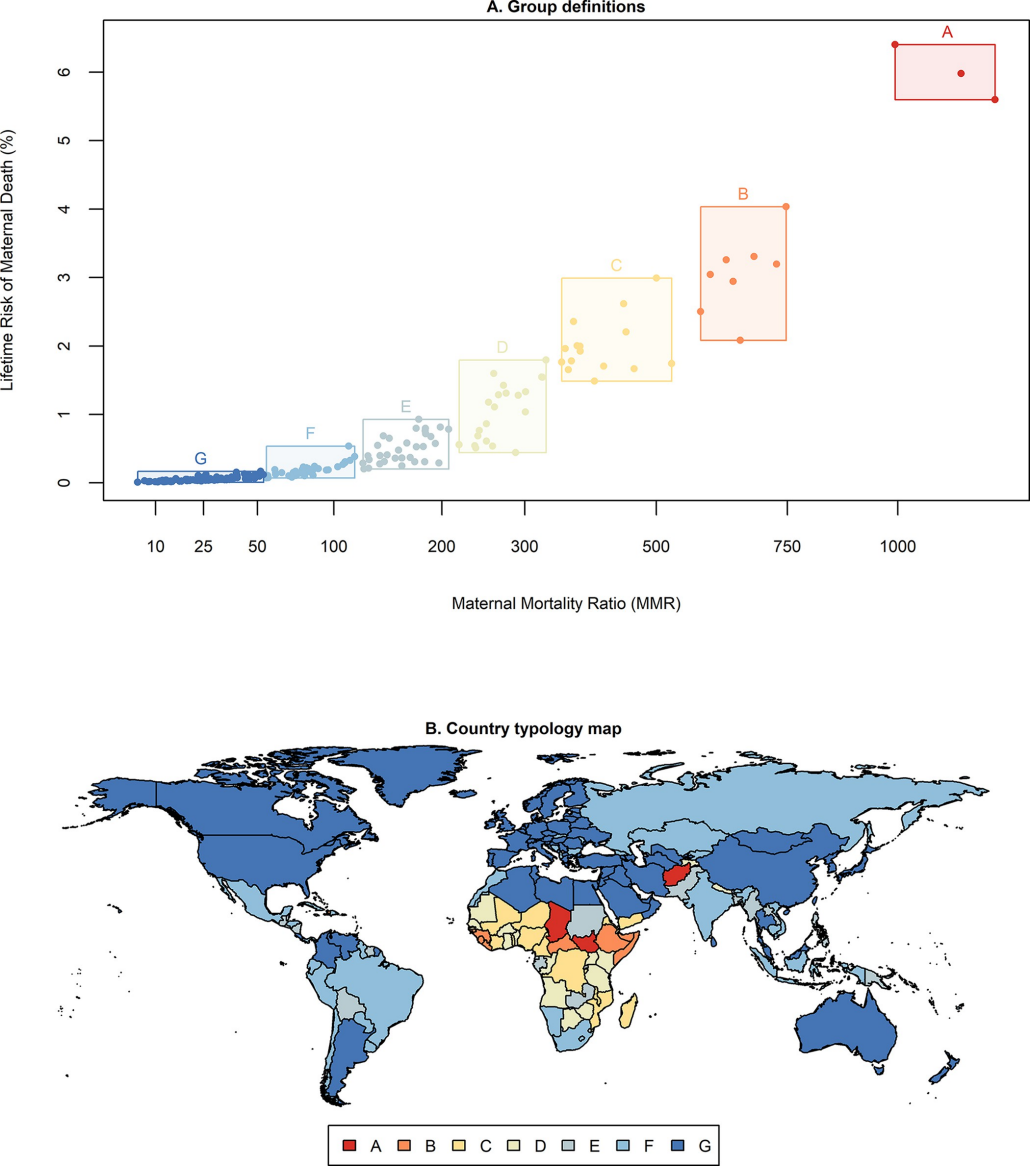
Maternal health country typology groups. (A) Group definitions: Maternal mortality ratio (MMR) + Lifetime risk of maternal death (LTR). (B) Country typology map. Source: The map was plotted by the authors in R using the ‘maps’ package v3.4.1, based on public domain boundaries (1:50m) from the Natural Earth data project (https://www.naturalearthdata.com/about/terms-of-use/).

We identified 7 groups of country clusters by MMR and LTR (Fig 1A), and find that these groupings include countries from different geographical regions (Fig 1B). Table 2 reports the summary characteristics for each group, such as maternal deaths and TFR, and lists the highest burden countries (up to 10) in each group by maternal deaths. We find that each of these groups has different salient features.

**Table 2:** Maternal health indicators (2022) and policy impacts by country group.

	Group Means		Other Maternal Indicators					Policy Impact: Projected Percent Reduction in Maternal Deaths 2030–2050				Countries	
Group	MMR	LTR (%)	Maternal Deaths	TFR	CPR %	Facility Births %	C-Section Rate %	Family planning	Community-based + Linkages to care	Facility births	Quality of care	#	Highest Burden
A	1174	5.85	32,000	4.53	24.1	50.3	2.5	49.5	18.3	22	13	3	Chad, Afghanistan, South Sudan
B	647	2.98	51,700	4.52	31.1	36.8	2.4	16.9	27.9	51.5	15.1	8	Ethiopia, Guinea, Somalia, Sierra Leone, Liberia, Haiti, Central African Republic, Guinea-Bissau
C	435	2.39	113,200	5.20	27.2	66.4	4.4	7.3	12.4	22.6	21.9	15	Nigeria, DRC, Niger, Mozambique, Madagascar, Cameroon, Mali, Cote d’Ivoire, Yemen, Burundi
D	288	1.29	43,100	4.15	37.0	75.6	9.2	9.8	18.8	30.5	19.7	21	Tanzania, Uganda, Angola, Kenya, Burkina Faso, Ghana, Senegal, Benin, Nepal, Zimbabwe
E	151	0.43	30,800	2.81	50.3	73.5	21.4	5.5	14.9	24.1	18.7	32	Pakistan, Philippines, Bangladesh, Sudan, Zambia, Rwanda, Papua New Guinea, Myanmar, Guatemala, Bolivia
F	78	0.18	50,300	2.26	51.0	92.2	27.8	11.1	13.1	9.7	4.5	35	India, Brazil, Vietnam, Indonesia, Mexico, South Africa, Russia, Morocco, Peru, Honduras
G	25	0.05	17,900	1.87	69.0	99.1	41.9	11.8	5.6	2.7	1.7	86	China, Egypt, USA, Iraq, Algeria, Syria, Iran, Turkey, Venezuela, Colombia

Indicators are weighted by population size for each group, except for Maternal Deaths which is the sum.

MMR: Maternal mortality ratio (number of maternal deaths per 100,000 live births), LTR: Lifetime risk of maternal death, TFR: Total fertility rate, CPR: Contraceptive prevalence rate, Facility Births: Percentage of births which occur in a health facility, C-Section Rate: Percentage of deliveries that occur via caesarean section (both elective and emergency).

Highest Burden Countries refers to countries with the largest number of maternal deaths in each group (up to 10 are displayed).

Group A consists of only three countries (Chad, Afghanistan, South Sudan) which are the highest burden countries for both MMR (over 1,000) and LTR (around 6%). These three countries accounted for an estimated 32,000 maternal deaths in 2022 (9% of the global total), and as outliers highlight the impact that legacies of war, violence, and political instability have on women’s health in particular. The average TFR for women in this group is 4.53, and we find that improving access to family planning for this group is projected to yield the largest reductions in maternal deaths among the policies assessed, preventing nearly 50% of the projected maternal deaths between 2030–2050. The next most effective policy identified was increasing the proportion of facility births, which is projected to prevent 22% of maternal deaths between 2030–2050.

Group B includes countries such as Ethiopia, Guinea, and Haiti, which still have high burdens of maternal mortality, but as a group have mean MMR and LTR estimates nearly twice as low as the outliers in Group A. Group B is estimated to account for 51,700 maternal deaths in 2022 (15% of the global total). Within this group, countries have similar MMR estimates but more variation in LTR, highlighting the impact of differences in fertility levels among countries with similar levels of obstetric risk. The highest priority in these countries is increasing facility births, which is estimated to prevent 51.5% of projected maternal deaths between 2030–2050, followed by improving community-based care and linkages to facility care (27.9% reduction in maternal deaths).

Group C overlaps with Group B in terms of LTR, but countries in this group are estimated to have lower obstetric risk (as estimated via MMR), including countries with large numbers of total maternal deaths such as Nigeria and the Democratic Republic of Congo. This group is estimated to account for 113,200 maternal deaths in 2022 (33% of the global total) and is the group with the largest global burden in terms of total maternal deaths. The highest priority in this group among the policies assessed is increasing facility births and improving quality of care, which yield very similar projected reductions in maternal deaths between 2030–2050 (22.6% and 21.9%, respectively).

Countries in Group D have MMRs around 300 with LTRs below 2%. This group includes countries such as Tanzania, Uganda, Angola, and Nepal, and accounts for an estimated 43,100 maternal deaths in 2022 (13% of the global total). In group D we find that countries with similar levels of MMR can have large variations in LTR (over 2-times differences), which reflects differences in underlying fertility and thus exposure to obstetric risk. The policies with the largest projected impact on maternal deaths between 2030–2050 for this group are increasing facility births (30.5% reduction) and improving quality of care (19.7% reduction).

Many countries in Group E still have estimated MMR levels above the SDG country target of 140, but as a group they are close to meeting this target. This group includes countries such as Pakistan, the Philippines, and Bangladesh, and accounts for an estimated 30,800 maternal deaths in 2022 (9% of the global total). Similar to Groups C and D, the highest estimated policy impacts for this group are increasing facility births (24.1% reduction in maternal deaths between 2030–2050) and improving quality of care (18.7% reduction).

Group F includes countries with estimated MMRs in 2022 below the SDG country target of 140, such as India, Brazil, Vietnam, and Indonesia. This group accounts for an estimated 50,300 maternal deaths in 2022 (15% of the global total). The estimated proportion of facility births in 2022 for women in this group was over 90%, so the scale-up policies with the largest projected impact on maternal deaths between 2030–2050 are instead improving community-based care and linkages to care for women who deliver at home (13.1% reduction) and improving access to family planning (11.1% reduction).

Group G includes countries with the lowest MMRs and LTRs, and includes large countries such as China, Egypt, and the United States. This group accounts for 17,900 maternal deaths in 2022 (5% of the global total). Although these countries in general have relatively low burdens of maternal mortality, there is heterogeneity in this group. For example, we see that countries with low LTRs can have variable MMRs, ranging from <10 to over 50, due to different combinations of low obstetric risk and/or low fertility. The policies for this group that yield the largest projected reduction in maternal deaths between 2030–2050 are improving access to family planning (11.8% reduction) and improving community-based care and linkages to care for high-risk women (5.6%), with over 99% of women in this group estimated to have given birth in health facilities in 2022.

## Discussion

We developed a maternal health country typology framework based on recent estimates of MMR and lifetime risk of maternal death (LTR), accounting for levels of both obstetric risk and fertility which impact the cumulative risk of repeated pregnancy exposure. Previously, countries have been grouped by characteristics such as income and geography, which often make sense for various reasons, but may not be useful for guiding policies specific to maternal health.

A theoretical obstetric transition framework has previously been developed to explain gradual changes that countries may experience as they make progress in eliminating avoidable maternal mortality [6]. Although multiple indicators have been explored in conjunction with this framework, the obstetric transition phases are defined using MMR alone, which is an indicator of obstetric risk per pregnancy, and groups countries into levels of avoidable maternal deaths. For example, Stage I is defined as countries with high MMRs (>1000), where the majority of women experience situations close to the natural history (i.e., the absence of medical interventions) of pregnancy and childbirth, with Stage V defined as the most advanced level where all avoidable maternal deaths are actually prevented, a largely theoretical stage with very low MMRs (e.g., <5) [6].

The obstetric transition framework has been used to assess specific countries and identify priorities for reducing maternal mortality based on the main determinants of mortality at each stage, with country priorities sometimes deviating from their assigned stages due to local contextual factors such as health system weaknesses and barriers to care [9]. As a specific country example, a recent analysis focused on Somalia estimated that it has moved from stage I to stage II as a result of improvements in the utilization of maternal health interventions [10]. Another recent review groups countries into these stages based on estimated trends in maternal mortality, and discusses the socio-economic and health determinants that influence maternal health across different stages, emphasizing the need for multi-faceted strategies to improve global maternal health [11].

However, although the need for comprehensive strategies is acknowledged, the obstetric transition framework considers only one maternal health indicator (i.e., MMR), which provides a limited view of country differences and progress, regardless of the indicator chosen. Another study examines trends in indicators for maternal, stillbirth, and neonatal deaths, assessing how causes of death and fertility rates change across different phases [12]. Comparing multiple indicators it highlights the interconnectedness of mortality determinants for mothers and babies and the need for integrated approaches to address these issues comprehensively [12].

In our analysis on maternal health we extend the sole focus on MMR to also consider lifetime risk of maternal death (LTR), which is sensitive to changes in both fertility and obstetrical risk (i.e., the risk associated with a single pregnancy and childbirth) and can be used to assess cumulative risk of repeat pregnancy. Together, these two continuous indicators provide an intuitive, simple way to group countries and also track trends over time. Using our typology framework we group countries using recent estimates (2022) from the GMatH model [3]. Our country typologies also find a strong gradient by MMR, but are based on clustering analysis of empirical estimates instead of specific theoretical thresholds. Although recent estimates of MMR and LTR are generally correlated across countries globally, the relationship between these indicators may change over time, and can vary widely within country groups. As maternal health changes in countries over time, this framework can be used to track trends and identify (potentially new) groupings of countries.

Using recent estimates, we identified 7 groupings of countries with different salient features, ranging from countries with high MMR and LTR (high burden countries: A-B), countries with low MMR and LTR (low burden countries: F-G), and a range of countries with middle levels of burden (C-E). Indeed, these typologies provide an alternative way to assess “burden”, which is often evaluated using total maternal deaths, which is an informative indicator but can be driven by countries with large population sizes, such as India and Nigeria [3]. Using this typology we find that “high burden” countries (A-B) account for nearly 25% of global maternal deaths, with Group A accounting for nearly 10% of the global total from only 3 countries.

We find that there are large differences in these typology indicators (MMR and LTR) across country groups, with each group having its own salient features. However, we find that there can also be substantial differences in these indicators within country groups, highlighting the importance of considering a dashboard of multiple indicators when assessing progress in maternal health.

Looking at other maternal health indicators, we find general trends in the total fertility rate (TFR) and contraceptive prevalence rate (CPR) by country groups, but with no clear monotonic trend for these indicators among the highest burden countries. For example, although the estimated TFR generally declines from Group A (4.53) to Group G (1.87) the average TFR for women in Group C is estimated to be the highest (5.2). This highlights the importance of health system strengthening efforts to make pregnancy safer and reduce obstetric risk, regardless of the number of births experienced by women in the population. We also find a general increasing trend of facility births and c-section rates by decreasing country burden, but with wide variations at the country level, and likely large disparities in access for women within countries.

Using our typology analysis in which we clustered countries into groups based on recent estimates of MMR and LTR, we estimated the impact of different policy interventions for each group, which can help inform policy guidance in different settings and better contextualize discussions around global maternal health. We find that reducing fertility in the highest burden countries (Group A) is estimated to yield the largest impact among single policy interventions, followed by increasing the number of women who deliver in facilities. In the next highest burden group (Group B), we find increasing facility births is estimated to yield the largest benefit, followed by improving community-based linkages to care. In low burden countries (F-G) we find that family planning and improving community-based linkages to care are also estimated to yield the highest impact compared to current trends. Although the ordinal ranking of policy impacts is the same for Groups C-E, the absolute impact of these polices differs among these groups. For Group C, the impact of increasing facility births is nearly tied with improving quality of care, whereas for Group D increasing facility births has a far larger effect (over 10 percentage points higher than improving quality of care), while for Group E the difference is smaller (around 5 percentage points difference).

Interestingly, we find an inverse “U-shaped” relationship between country burden group and the impact of improving quality of care, with the largest impact projected to occur among middle burden countries (groups C-E). This is not because quality of care does not need to be improved in high burden groups (A-B), but the relative importance of family planning and ensuring women have access to facility-based care at all are more immediate priorities in these settings. Although improving quality of care will be critical in all settings, there is limited value in quality of care improvements if many women do not have access to facility-based care. Conversely, in low burden countries (F-G), because quality of care is already relatively high, improving family planning and linkages to care, especially for high-risk women, are more immediate priorities. Quality of care improvements are estimated to yield benefits for all country groups, but the interdependence of health system factors means that improving access to care will also be necessary in settings where many women deliver at home.

Ideally, countries could simultaneously improve access to care and the quality of care received to avoid a repeat of the lessons learned as part of the Janani Suraksha Yojana program, a conditional cash-transfer program to incentivize facility delivery in India which succeeded in increasing facility births but had no significant impact on maternal mortality due to a lack of focus on quality improvements [13,14]. Similar insights were found in an analysis of large population-based cluster-randomized controlled trials in Ghana, which found that facility birth does not necessarily improve survival for women or infants if quality of care is not addressed [15]. In addition, perceptions of facility quality has been identified as a factor in women’s decision-making around where to deliver, with women often bypassing facilities in search of quality services [16–18], highlighting the importance of considering women’s preferences and decision-making ability when designing and implementing policy interventions to improve maternal health.

Although our typology analysis provides a useful framework and general insights, further contextualization and analysis at the country level is needed to guide specific priority-setting and planning decisions, especially as the feasibility of proposed interventions may vary across diverse contexts. Similarly, it is also important to consider equity within countries, as subgroups within countries may have very different maternal health outcomes [19], with different typologies likely existing within the same country.

Although our typologies are based on empirical estimates, there is often large uncertainty around the maternal indicator values (MMR and LTR) used to group countries, and how these indicators may be changing over time. We grouped countries based on the mean estimated value of each indicator, but acknowledge the large uncertainty around these estimates (previously characterized) [3], differences in which could result in some countries being assigned to different groups. Nevertheless, although these indicator estimates are sometimes subject to large uncertainty, the general position of countries across these groups (i.e., high burden vs low burden) is likely robust to this uncertainty, and this approach provides a simple, useful framework for policy guidance.

Overall, our typology analysis based on MMR and LTR estimates allows countries to be grouped into clusters based on similar levels of obstetric risk and fertility levels, accounting for cumulative risks of repeated pregnancy exposure. We find that these maternal health indicators vary widely across groups, and also vary within groups, highlighting the importance of considering multiple indicators when assessing progress in maternal health. The impact of policy interventions also differs by country typology, with different strategies estimated to yield the largest impact in different contexts. These findings provide stakeholders (e.g., local and international policymakers, funders, researchers) with information to help prioritize interventions to accelerate progress in maternal health.
